# Tissue Classification of Breast Cancer by Hyperspectral Unmixing

**DOI:** 10.3390/cancers15102679

**Published:** 2023-05-09

**Authors:** Lynn-Jade S. Jong, Anouk L. Post, Dinusha Veluponnar, Freija Geldof, Henricus J. C. M. Sterenborg, Theo J. M. Ruers, Behdad Dashtbozorg

**Affiliations:** 1 Department of Surgery, Netherlands Cancer Institute, Plesmanlaan 121, 1066 CX Amsterdam, The Netherlands; 2 Department of Nanobiophysics, Faculty of Science and Technology, University of Twente, Drienerlolaan 5, 7522 NB Enschede, The Netherlands; 3 Department of Biomedical Engineering and Physics, Amsterdam University Medical Centers, University of Amsterdam, Meibergdreef 9, 1105 AZ Amsterdam, The Netherlands

**Keywords:** breast-conserving surgery, hyperspectral imaging, resection margin assessment, breast tissue, hyperspectral unmixing, tissue classification

## Abstract

**Simple Summary:**

To minimize the risk of cancer recurrence, it is crucial for surgeons to assess the resection margins (surface) of surgical specimens during breast-conserving surgeries to determine whether the tumor has been removed entirely. However, this is often not easy and also current techniques lack to aid the surgeons. In our study, we used a hyperspectral imaging technique to overcome this challenge. To assess the resection margins with hyperspectral imaging, a classification model should be developed, which requires a dataset with accurate ground-truth labels. Since it is difficult to establish such a dataset, we introduced a novel approach based on hyperspectral unmixing to enable an accurate correlation between hyperspectral measurements and histology ground-truth labels. Subsequently, we developed a classification model for tumor tissue detection on the lumpectomy resection surface of 189 patients. We achieved a sensitivity of 94% and a specificity of 85%, which demonstrated the potential of hyperspectral imaging for breast-conserving surgeries.

**Abstract:**

(1) Background: Assessing the resection margins during breast-conserving surgery is an important clinical need to minimize the risk of recurrent breast cancer. However, currently there is no technique that can provide real-time feedback to aid surgeons in the margin assessment. Hyperspectral imaging has the potential to overcome this problem. To classify resection margins with this technique, a tissue discrimination model should be developed, which requires a dataset with accurate ground-truth labels. However, establishing such a dataset for resection specimens is difficult. (2) Methods: In this study, we therefore propose a novel approach based on hyperspectral unmixing to determine which pixels within hyperspectral images should be assigned to the ground-truth labels from histopathology. Subsequently, we use this hyperspectral-unmixing-based approach to develop a tissue discrimination model on the presence of tumor tissue within the resection margins of ex vivo breast lumpectomy specimens. (3) Results: In total, 372 measured locations were included on the lumpectomy resection surface of 189 patients. We achieved a sensitivity of 0.94, specificity of 0.85, accuracy of 0.87, Matthew’s correlation coefficient of 0.71, and area under the curve of 0.92. (4) Conclusion: Using this hyperspectral-unmixing-based approach, we demonstrated that the measured locations with hyperspectral imaging on the resection surface of lumpectomy specimens could be classified with excellent performance.

## 1. Introduction

With an estimated number of 2.3 million new diagnoses in 2020, breast cancer is the most prevalent type of cancer among women worldwide [1]. Treatment of this disease often consists of breast-conserving surgery combined with adjuvant radiotherapy [2,3]. During breast-conserving surgery, surgeons aim to remove malignant tumor tissue with a small border of healthy tissue around it, called a resection margin, while sparing the remaining healthy tissue of the breast. However, it is not always easy to distinguish the border between healthy and tumor tissue during surgery. Consequently, in up to 36% of surgeries [4], the margin of the resected breast tissue still contains tumor tissue, which is described as a tumor-positive resection margin. As the latter indicates a larger risk of recurrent breast cancer [5,6], the patient is subjected to additional treatment, e.g., a re-operation or an extra radiotherapy boost, which evidently affects the cosmetic result, the patient’s quality of life, and the treatment costs [7,8]. Currently, resection margin assessment is performed by histopathological analysis, which could take between 3 and 5 days after surgery. To improve breast-conserving surgeries, there is a need for a margin assessment technique that allows real-time feedback during surgery regarding the presence of tumor tissue on the resection margins of the surgical specimen.

Hyperspectral imaging (HSI) is a technique that has shown promising results for the discrimination between tumor and healthy tissue in the breast [9,10,11,12,13,14], as well as in the colon [15,16], ovary [17], thyroid [18,19], and skin [20,21]. Hyperspectral imaging is a diagnostic technique in which digital imaging is combined with spectroscopy. Each pixel within the captured image contains the reflected intensity for a wide range of colors, known as a spectrum. These spectra can be considered an ‘optical fingerprint’, since they are influenced by the tissue composition. Compared to a normal color image, which only has three spectral bands corresponding to the visual primary colors red, green, and blue, a hyperspectral image thus contains a large number of spectral bands to yield more detailed information about the sample’s optical properties. A main advantage of hyperspectral imaging is that it can image tissue in a quick, non-invasive, and harmless manner.

Hyperspectral imaging has been shown to be able to differentiate healthy from malignant tissue in gross-sectioned breast tissue slices using supervised classification methods [9,10,11]. In a study by Kho et al., promising results were achieved with a sensitivity and specificity of, respectively, 98% and 99% for discriminating tumor tissue from healthy tissue in breast tissue slices [10]. This demonstrated the potential of hyperspectral imaging for margin assessment. Even so, a tissue classification algorithm developed on tissue slices cannot be directly used for intact resection specimens. Previous studies demonstrated differences between slices and specimens in terms of tissue thickness, freshness, surface structure, blood saturation, and cauterization, which in turn can influence the measured spectra within the hyperspectral images [11,22]. Therefore, we recently took the next step towards clinical translation by developing an algorithm specifically for breast cancer resection specimens [11].

In that study, we discussed the challenge of establishing ground-truth labels when developing classification algorithms for resection specimens. In the standard histopathology processing of resection specimens at the Netherlands Cancer Institute—Antoni van Leeuwenhoek hospital, a specimen was first cut into thick slices and then a very thin slice (±4 μm) was stained with hematoxylin and eosin (H&E) and inspected under a microscope by the pathologist. For our study, this resulted in a tissue label for that entire H&E slide (e.g., healthy or tumor). After histopathology processing and examination, the next step for us was to identify which pixel in the hyperspectral image should be assigned to this ground-truth label and should be used to develop a classification algorithm. One often-used approach to correlate the H&E slide to the hyperspectral image is to place an ink mark (or other types of marker) on the surface of the specimen which is visible in both the camera image and the H&E slide. An H&E slide is usually made perpendicular to the surface of the specimen, and since the ink mark generally covers an area of approximately 5 by 5 mm, while an H&E slide is only about 4 μm thick, there is always ambiguity in deciding which of the pixels covered by the ink mark corresponds best to the H&E slide. This ambiguity can be especially problematic in inhomogeneous tissue such as resection specimens, where neighboring pixels covered by the ink mark can contain different tissue types. The classification performance of a machine learning model strongly depends on the accuracy of the ground-truth labels. Therefore, in order to train a robust classification model, it is crucial to ensure that the ground-truth labels truly correspond to the pixels from which the spectra are used in the development of the algorithm.

In our previous study, we assumed that the H&E slide always came from the center of the ink mark. Therefore, we assigned the pathology labels to the pixel in the center of the patch of pixels containing the ink mark. However, in practice, it is impossible for a histopathologist to slice the tissue with such precision that the H&E slide truly is taken from the center of the ink mark. In the current study, we propose a novel method based on hyperspectral unmixing to decide which pixel (covered by a mark) in a hyperspectral image should be assigned the ground-truth label from histopathology, and should subsequently be used in training a classification algorithm. We apply this approach to breast cancer resection specimens and show that our novel method improves the classification performance compared to using the center pixel approach. While the current study focuses on breast cancer, the approach we developed based on hyperspectral unmixing can potentially also be used for any other type of tissue classification algorithm. To the best of our knowledge, this is the first study that has used such an approach to assign pathology labels.

## 2. Materials and Methods

### 2.1. Study Design

In the period from 2018 to 2021, lumpectomy specimens were included from 200 patients that received primary breast-conserving surgery (either with or without neoadjuvant therapy) at The Netherlands Cancer Institute—Antoni van Leeuwenhoek hospital. These lumpectomy specimens were imaged with hyperspectral cameras immediately after resection, before pathology processing. This ex vivo study complied with the Declaration of Helsinki and was approved by the Institutional Review Board of The Netherlands Cancer Institute—Antoni van Leeuwenhoek hospital. Based on the Dutch medical research involving human subjects act (WMO), no informed consent from the patients was required.

### 2.2. Hyperspectral Imaging

**Figure 1:**
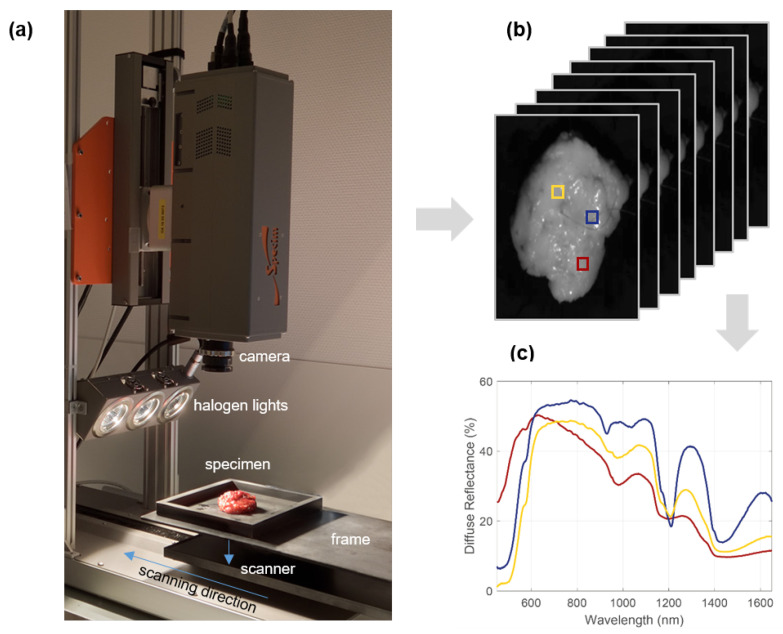
Hyperspectral imaging setup. To allow reproducible measurements, the lumpectomy specimen is positioned with a container upon a frame which fits the scanner (**a**). By moving the scanner under the camera, a 3D hypercube of the specimen can be acquired with spatial information along the *x* and *y* axes and spectral information along the *z* axis (**b**). For each pixel in the hypercube, an individual spectrum can be obtained which contains information about the specimen’s optical properties; example spectra corresponding to boxes depicted in yellow, blue, and red are shown in (**c**).

The setup consisted of 3 halogen light sources (2900 K) to illuminate the sample from a 35 degree angle, and a scanner to move the sample under the camera enabling it to be imaged line-by-line, see Figure 1. The acquired 3D data structure is called a hypercube, which contains both spatial information (*x* and *y* dimensions) and spectral information (*z* dimension).

To cover a broad wavelength range, two pushbroom hyperspectral cameras (Specim, Spectral Imaging Ltd., Oulu, Finland) were used. The first camera (PFD-CL-65-V10E, CMOS sensor 1312×384 pixels, 384 wavelength bands, 3 nm increments, 0.16 mm/pixel) operates in the visual (VIS) region between 400 and 1000 nm, and the second camera (VLNIR CL-350-N17E, InGaAs sensor 320×256 pixels, 256 wavelength bands, 5 nm increments, 0.5 mm/pixel) in the near-infrared (NIR) region between 900 and 1700 nm (the latter is depicted in Figure 1 but the setup of both cameras is similar). The cameras were controlled with data acquisition software (LUMO v2016-427, Specim, Spectral Imaging Ltd., Oulu, Finland).

Considering the specimen as a cube with six resection sides, each side was imaged. For acquiring the HSI hypercube for each side, first, the specimen was positioned on a container tray which can be fixated to the scanner translation frame. This enabled us to acquire the hyperspectral images for each resection side with both cameras in sequence without manipulating the specimen when moving the translation frame from one camera to another. After one resection side was imaged, the specimen was repositioned manually so that the following side could be imaged again with both cameras in sequence.

To account for the dark current in the hyperspectral imaging systems and for the spectral variations in the illumination setup, we converted the raw intensity data into calibrated diffuse reflectance images as described in [9]. The hyperspectral images of the camera systems differ in terms of size and spatial resolution. Therefore, we resized the images to 320×256 pixels using an affine registration and matched the spatial resolution (0.5 mm/pixel). We excluded the wavelength bands at the extremities of the spectral ranges of the hyperspectral cameras (because of the sensors’ low spectral sensitivity at the extremities), which resulted in a hyperspectral image with a wavelength range from 450 to 951 nm (VIS camera, 318 wavelength bands) to 954–1650 nm (NIR camera, 210 wavelength bands). Both hyperspectral imaging systems included an individual suspension system with corresponding light sources. Therefore, the illumination angle of the systems could slightly differ and may cause a bias (discontinuity) between the spectra of the hyperspectral cameras. We accounted for this by first calculating the difference in diffuse reflectance between the first wavelength (954 nm) of the NIR camera and the last wavelength (951 nm) of the VIS camera for each spectrum. Subsequently, this difference was added to the entire spectrum of the NIR camera to connect the spectra of both cameras. Additionally, a standard normal variate (SNV) normalization was applied to account for surface reflections and the uneven tissue surface of the lumpectomy specimen, using the following:

(1) $$R_{SNV}\left(\lambda \right)=\frac{R_{cal}\left(\lambda \right)-\mu }{\sigma }$$

where $R_{cal}\left(\lambda \right)$ is the calibrated reflectance spectrum, μ is the mean of $R_{cal}\left(\lambda \right)$, and σ is the standard deviation of $R_{cal}\left(\lambda \right)$ [9,23].

### 2.3. Pipeline to Assign Ground-Truth Labels

**Figure 2:**
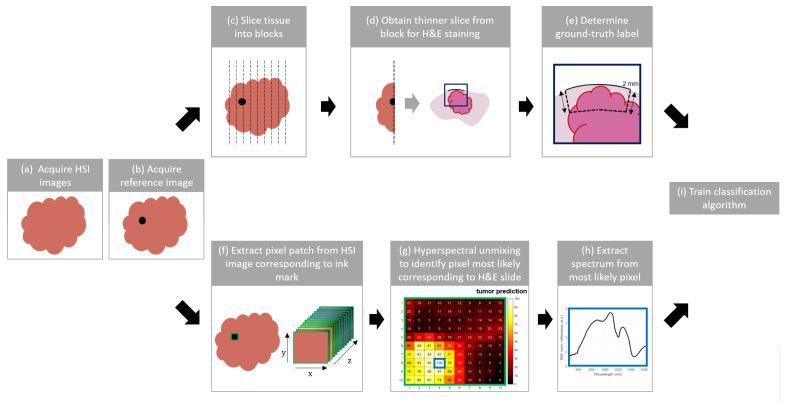
Pipeline to train a tissue classification algorithm where ground-truth labels are assigned to the training set based on hyperspectral unmixing. (**a**) First, the hyperspectral images of the specimen are acquired. Subsequently, black ink marks are placed on the specimen and a reference image (**b**) is captured to correlate the hyperspectral images to the histology slides. The upper pipeline shows the histopathological workflow, where (**c**) the lumpectomy specimen is gross-sectioned into tissue blocks of a few millimeters thick; (**d**) a slice of ±4 micrometers thick is sliced from this block and (**e**) stained with H&E, digitized and inspected by a pathologist who delineates the malignant tissue region in red up to a depth of 2 mm below the inked surface. The lower pipeline shows the workflow to determine which pixel covered by the ink mark should be assigned to this ground-truth label. (**f**) A patch of 10 by 10 pixels is extracted for each ink mark; (**g**) based on hyperspectral unmixing a tumor prediction map is created for this patch. (**h**) This map is then used to select the spectrum from the pixel that most likely corresponds to the ground-truth label which is used to (**i**) train the classification algorithm.

We introduce a pipeline to develop a tissue classification algorithm where ground-truth labels are assigned to the training set based on hyperspectral unmixing (Figure 2). Our new method based on hyperspectral unmixing addresses the ambiguity in deciding which pixel (and thus which spectrum) should be assigned to the ground-truth label obtained from histopathology.

#### 2.3.1. Reference Image

After the hypercubes were obtained (Figure 2a), black ink marks of approximately 5 × 5 mm were placed on the resection specimen to correlate the hyperspectral images to the histology slides. Subsequently, a reference image was taken with the VIS camera to determine which pixels in the hypercubes correspond to the black ink marks (Figure 2b). Due to limitations in the histopathology workflow, only a maximum of three ink marks could be placed on a single side of the specimen and be correlated with the ground-truth hematoxylin and eosin (H&E) stained images. To increase the chance that our dataset contained enough tumor data, tumor-suspicious areas were identified by visual inspection and palpation as well as an ultrasound scanner, and at least one of the ink marks was placed on the tumor-suspicious area. It should be noted that the ink mark locations were selected prior to the pathology processing, which means that there was no certainty regarding the tissue type underneath the ink marks.

#### 2.3.2. Pathology Processing

After the hyperspectral images were obtained, the lumpectomy specimens were processed at the pathology department. First, the specimens were sliced into blocks of a few millimeters thick (Figure 2c). Next, from each block that contained a black ink mark, one thinner slice was made (±4 μm) and stained with hematoxylin and eosin (Figure 2d). This H&E slice was digitized, and malignant tissue regions (i.e., carcinoma in situ or invasive carcinoma) were annotated by a pathologist. To discriminate between fat and connective tissue in the remaining healthy tissue, we used a threshold value of 0.90 for the intensity within the green channel of the digitized H&E image so that, based on the H&E color distribution, a distinction was made between fat (white) and connective tissue (pink). To determine the ground-truth label for the H&E image, an area up to 2 mm underneath the black ink surface was selected (Figure 2e). If any carcinoma in situ (CIS) or invasive carcinoma (IC) was present within this area, the label malignant was assigned, whereas in the case of only fat or connective tissue, the label healthy was assigned.

#### 2.3.3. Assigning Ground-Truth Labels with Hyperspectral Unmixing

Hyperspectral unmixing can be used to estimate the fractional percentages (abundances) of pure components (endmembers) within spectra (pixels). In this study, we use hyperspectral unmixing to estimate the fractional abundances of malignant tumor and healthy tissue within the spectra from the pixels covered by the ink marks.

We performed hyperspectral unmixing using a linear mixing model (LMM). An LMM assumes that each detected photon has interacted with only one tissue type and that photons from different tissue types are mixed in the camera as a result of its low spatial resolution [24]. Since hyperspectral imaging detects diffuse light that has traveled a few millimeters through tissue, and breast tissue is inhomogeneous, the assumption that detected photons have interacted with only one tissue type is likely violated. Therefore, nonlinear mixing models represent a more realistic scenario for hyperspectral imaging. Even so, nonlinear mixing models often use complex deep learning methods, and thus generally require a large amount of data and computation time to unmix the data. On top of that, the most common nonlinear mixing models employ autoencoder architectures [25,26]. These unsupervised algorithms are more prone to extracting endmembers that are nonexistent, and thus the predicted fractional abundances might be incorrect and useless [26]. Since the number of labeled samples in our dataset is limited, we used a linear mixing model instead of a nonlinear mixing model.

To estimate the fractional abundances of malignant tumor and healthy tissue within the spectra from the pixels covered by the ink marks, we selected patches of 10×10 pixels (5 by 5 mm) and 528 wavelength bands (Figure 2f). Hereafter, we selected four endmembers for the different tissue components IC, CIS, fat, and connective tissue. Selection of the endmember spectra was performed according to the annotated H&E images that were correlated with the marked locations. For each endmember, a single H&E image was chosen, of which the area underneath the black ink mark only contained the respective tissue type. Subsequently, the center pixel of the associated marked location on the hyperspectral image was selected to obtain the endmember spectrum of this tissue component.

For each patch (measured location) on the lumpectomy resection surface, we created a map with the estimated abundances of malignant tissue (IC and CIS combined), see Figure 2g. With this map we intended to extract the spectra of the most relevant pixels per location. In the case of a malignant ground-truth label based on the H&E, the spectrum with the highest tumor prediction was extracted and labeled as tumor (Figure 2h), whereas in the case of a healthy ground-truth label, the spectrum with the lowest tumor prediction was extracted and labeled as healthy. Hence, using this prediction map, we could find the most representative spectra for each location.

The unmixing process described by the LMM is defined as

(2) $$X=\sum_{c=1}^{C}a_{c}e_{c}+\epsilon$$

where *X* is represented as a linear combination of the endmember spectra $e_{c}$ and their fractional abundances $a_{c}$ for each tissue class *c* and additional Gaussian noise ϵ. The model is subjected to the abundance non-negativity constraint as well as the abundance sum-to-one constraint. This means that $a_{c}$ should be ≥0 and the total sum of the fractional abundances should equal 1, respectively, [27,28,29]. *X* is the measured spectrum with $m_{\lambda }$ wavelength bands and $e_{c}$ can be extracted from ground-truth spectra. With these known variables, Equation (2) can be fitted to the measured spectrum with a constrained linear least-squares approach using the interior-point algorithm [30,31] if the total number of tissue classes *C* is <$m_{\lambda }$− 1, which is generally the case. The least-squares optimization is described by

(3) $${\left\{\hat{a}{}_{c}\right\}}_{LS}={\underset{{\left\{\hat{a}{}_{c}\right\}}_{c}}{\mathrm{argmin}}}_{}^{}{∥X^{\prime }\left({\hat{a}}_{1},...,\hat{a}{}_{c}\right)-X∥}_{2}$$

where $\hat{a}{}_{c}$ is the estimate for $a_{c}$ and X′ is the reconstructed spectrum. Thus, by inverting Equation (2) the unmixing problem can be solved with the estimation of the unknowns $a_{c}$ and ϵ as output.

Using the endmember spectra and the patch as input for the LMM, we could estimate the abundances of the tissue components for every pixel in the patch. Algorithm 1 shows a detailed explanation of this hyperspectral-unmixing-based approach.
**Algorithm 1** Label assignment based on hyperspectral unmixing     **Input:** Training samples H with labels L at locations *P*     **Output:** Certain Labeled representative spectra S, Ł 1:**for** i∈{1,2,3,...,N}**do**        ▷*N*: number of samples in training set 2:    **for**  $c\in \{1,2,...,T_{i}\}$ **do**             ▷$T_{i}$: number of ink points 3:        $M\leftarrow H_{i}\left(P_{cx}-5:P_{cx}+5:P_{cy}-5:P_{cy}+5\right)$   ▷extracting a patch 4:         U←LMM(M)      ▷ calculating tumor percentage based on unmixing 5:        **if** $L_{i}$ is 1 **then** 6:           $d\leftarrow \underset{d}{argmax}\left(U\right)$              ▷ the argument of the maxima 7:           $S_{i}\leftarrow P\left(d\right)$            ▷ extracting representative tumor spectrum 8:           Ł${}_{i}\leftarrow 1$                       ▷ assigning tumor label 9:        **else if** $L_{i}$ is 0 **then** 10:           $d\leftarrow \underset{d}{argmin}\left(U\right)$              ▷ the argument of the minima 11:           $S_{i}\leftarrow P\left(d\right)$           ▷ extracting representative healthy spectrum 12:           Ł${}_{i}\leftarrow 0$                       ▷ assigning healthy label 13:        **end if** 14:    **end for** 15:**end for**

### 2.4. Tissue Classification

**Figure 3:**
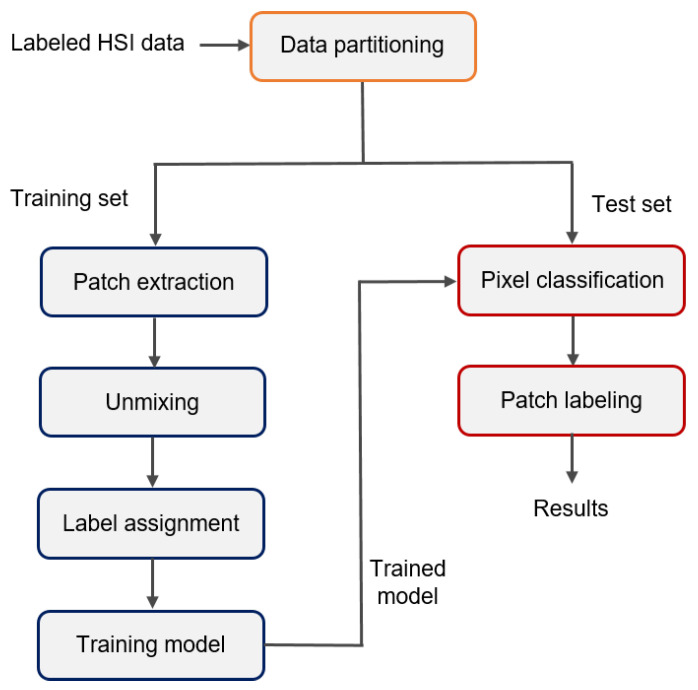
Proposed method to develop a classification algorithm where hyperspectral unmixing is used to determine which spectra should be assigned the ground-truth labels from histopathology for the training set.

Figure 3 outlines our approach to develop the classification algorithm. First, the dataset was split into an 80% training set and a 20% test set whilst the spectra were partitioned on a patient level. For each hyperspectral image in the training set, we extracted the patch of pixels (10 × 10 pixels) corresponding to the ink mark. Next, we used hyperspectral unmixing to select the pixel within the patch that most likely corresponded to the ground-truth label from histopathology. The spectrum from this pixel combined with the ground-truth label was then used to train the classification algorithm, for which we used a weighted kNN classifier, which is a supervised machine learning model. For the k-nearest neighbor classification model, the number of neighbors was set to 10 with the Euclidean distance metric and squared inverse distance weight.

For the test set, we also extracted the patch of pixels corresponding to the ink mark for each hyperspectral image. Next, we used the developed classification algorithm to classify each pixel within the patch individually. If minimally 10 out of the 100 pixels within the patch were classified as tumor, the patch was assigned the ground-truth label malignant (and healthy otherwise). This threshold was set to 10 pixels (pixel size ≈ 0.5 mm) as a re-excision is indicated when more than 4 mm of IC or a combination of IC with CIS is present on the resection surface [32].

#### Performance Testing

From a clinical perspective, it is particularly essential to distinguish healthy from malignant tissue. Therefore, we determined the number of measurement locations that were correctly classified as either healthy or malignant tissue. The prior is defined as the true negative (*TN*) rate, whereas the latter is defined as the true positive (*TP*) rate. Conversely, the false positive (*FP*) rate represents the number of measurement locations that were classified as malignant tissue which were healthy tissue, and the false negative (*FN*) rate is the number of measurement locations that were classified as healthy tissue which were malignant tissue. With these definitions, we calculated the sensitivity, specificity, and accuracy using the optimal cut-off point on the receiver operating characteristic (ROC) curve and the associated 95% confidence intervals (CI) according to the Clopper–Pearson interval method [33]. Furthermore, the area under the curve (AUC) and Matthew’s Correlation Coefficient (*MCC*) were determined. The latter is defined as

(4) $$MCC=\frac{TP\cdot TN-FP\cdot FN}{\sqrt{\left(TP+FP)(TP+FN)(TN+FP)(TN+FN\right)}}$$

with the *MCC* ranging from −1 (negative correlation) to +1 (perfect correlation). The *MCC* can be considered as a more robust alternative for accuracy in the case of an imbalanced dataset [34].

We compared our approach for assigning ground-truth labels in the training set to two other approaches: (1) selecting the spectra from the center pixels of each patch; (2) taking the average of all spectra within the patch. For each approach, the same partitioning of the data was used for the training and test sets to enable a fair comparison.

## 3. Results

### 3.1. Dataset Description

In total, the lumpectomy specimens of 189 female patients were included in the analysis. The remaining 11 patients (of the initially 200 patients measured) were excluded from the dataset because no tissue labels could be assigned to the measured locations, i.e., the ink marks could not be found on the H&E slides, and thus no correlation could be made with histopathology.

Table 1 provides an overview of the characteristics of the 189 included patients: the age, menopausal stage, breast side and density, type of neoadjuvant therapy, and the lumpectomy size. The age of the patients was 57 ± 11 years (mean ± STD), and most of them were in their post-menopausal stage. Based on the American College of Radiology (ACR) score, the largest group of patients had either a scattered fibroglandular or heterogeneously dense breast density. Furthermore, most patients did not have neoadjuvant therapy before the surgery. The size of the resected lumpectomy specimens was 57 ± 56 cm${}^{3}$ (mean ± STD).

Table 2 describes the distribution of healthy and malignant tissue in the lumpectomy dataset. The dataset consists of 151 patients in the training set (302 measured locations) and 38 patients in the test set (70 measured locations). The number of malignant tissue labels is lower compared to the number of healthy tissue labels because the measurement locations had been particularly chosen based on visual inspection and palpation before histopathological processing. Therefore, the exact location of the tumor tissue was not known at the time when measurement locations were selected.

### 3.2. Assigning Ground-Truth Labels with Hyperspectral Unmixing

**Figure 4:**
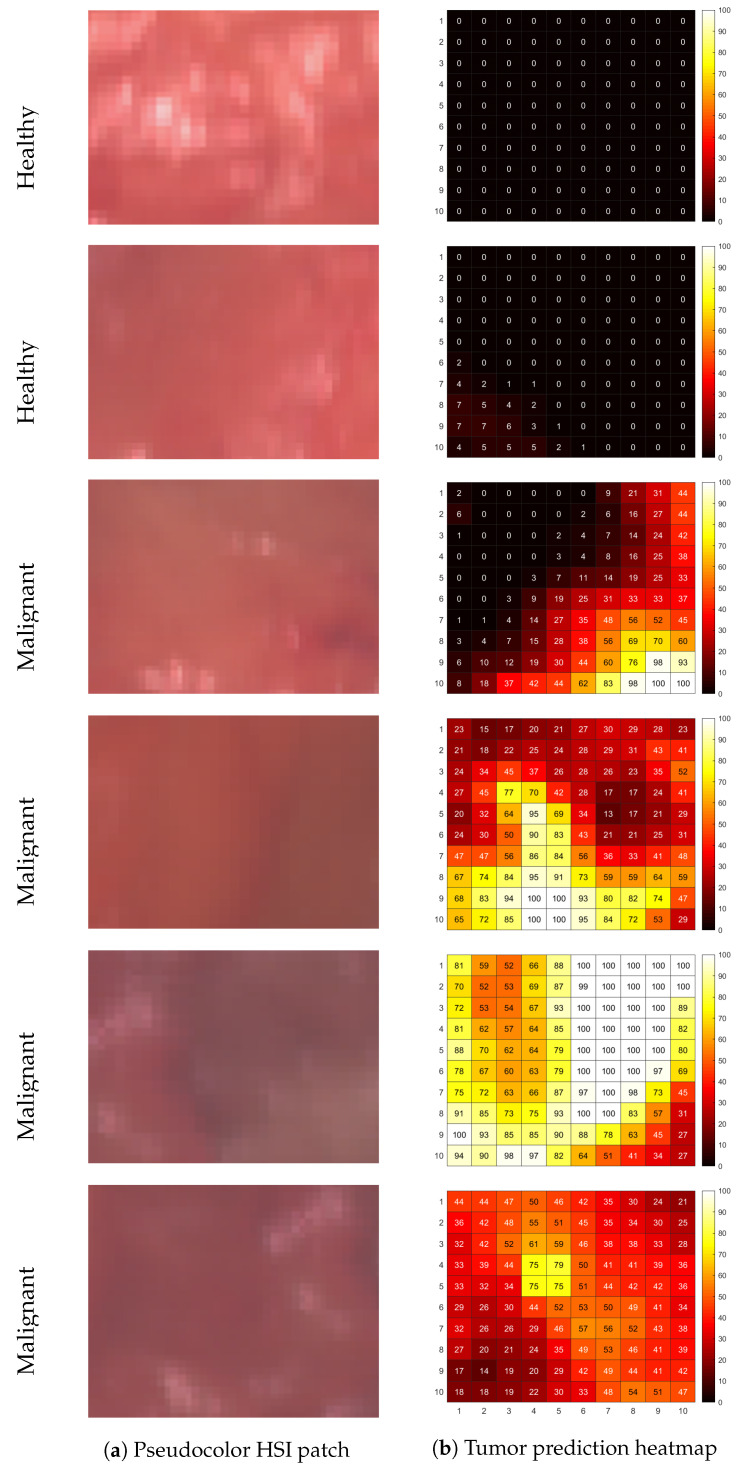
Tumor prediction map of different measured locations on the lumpectomy resection surface obtained with hyperspectral unmixing. (**a**) Pseudocolor image (wavelength bands at 649, 534, and 480 nm) based on the hyperspectral image with (**b**) the corresponding tumor prediction map. Rows 1–2: locations with a healthy tissue label from histopathology and a low tumor prediction. Rows 3–6: locations with a malignant tissue label and a high tumor prediction, despite how the tumor hotspots vary in terms of size and location.

Figure 4 illustrates examples of the tumor prediction maps of six measurement locations. These tumor prediction maps are the result of hyperspectral unmixing, and depict the estimated abundances of tumor tissue within each pixel. The first two rows of Figure 4 are from pixel patches that were assigned the ground-truth label ‘healthy’ based on the corresponding H&E slide. The tumor prediction map for the healthy pixel patches have very low estimated abundances of tumor tissue. The bottom four rows of Figure 4 are from pixel patches that were assigned the ground-truth label ‘malignant’. All four tumor prediction maps have a hotspot and a spatial gradient in the estimated abundances in the radial direction from high (bright) to low (dark) estimated abundances. It should be noted that the maximum value of the abundance of malignant tissue does not necessarily have to be 100% for a pixel patch belonging to an area that contains tumor tissue (bottom tumor prediction map). The tissue within the region of interest in the H&E slide (Figure 2e) is not necessarily homogeneous and can contain both malignant and healthy tissue. In the first three malignant patches there is much variation in the estimated abundances within the patch, and the hotspots (i.e., the pixels with the highest estimated tumor percentages) are not located in the center of the patches.

### 3.3. Tissue Classification

**Figure 5:**
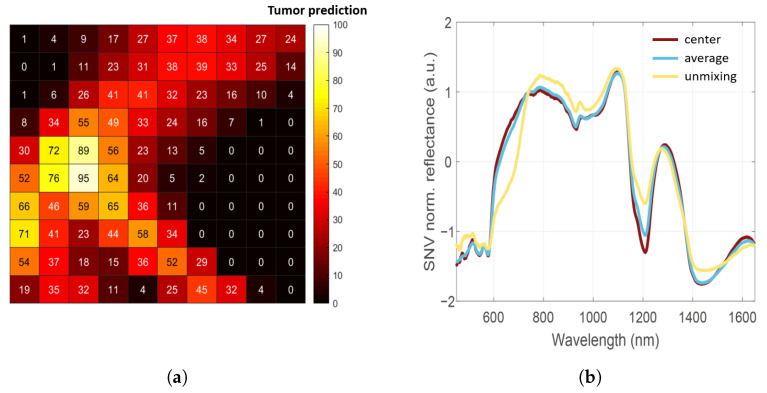
(**a**) Tumor prediction map for a pixel patch where the H&E slide was labeled malignant. The numbers are the estimated fractional abundances of tumor tissue obtained with hyperspectral unmixing. There is a tumor hotspot on the left, which suggests the tumor is not centered within the ink mark. (**b**) SNV normalized diffuse reflectance spectra of three different approaches for selecting pixels to assign ground-truth labels. There is a distinct difference in the SNV-normalized spectrum obtained with hyperspectral unmixing (pixel with the highest tumor value in the prediction map) compared to the center pixel and the average of all pixels in the patch.

Figure 5 illustrates the effect of the three different approaches for assigning ground-truth labels to spectra. The tumor prediction map (Figure 5a) implies that the tumor is not located in the center of the pixel patch. From the tumor prediction map it becomes clear that it will likely matter to which pixel within the patch the ground-truth label is assigned. Figure 5b shows an example of how different approaches (hyperspectral unmixing, center patch, or average patch) for selecting pixels to assign ground-truth labels will result in different spectra used to train the algorithm.

When comparing the SNV-normalized spectrum obtained with hyperspectral unmixing to the spectra of the center pixel and the average of all pixels in the patch, a distinct difference can be observed in the spectral shape. This spectral variability is mainly evident in the visual range from 600 to 730 nm, and in the near-infrared range around 1200 nm. Although the spectra of the center pixel and the average of all pixels vary slightly in intensity, they are mostly similar along the wavelength range.

**Figure 6:**
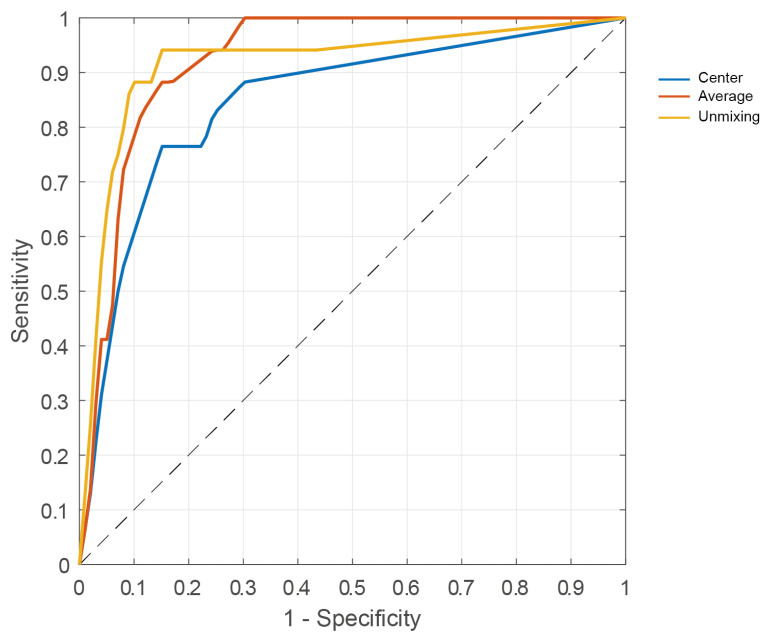
Comparison of the ROC curve of the hyperspectral-unmixing-based approach we developed (yellow line) to selecting the center pixel or taking the average of all spectra within a pixel patch.

Table 3 gives the performance metrics of the weighted kNN model for the discrimination between healthy and malignant tissue for each approach to select pixels and assign ground-truth labels. From Table 3 and the ROC curves shown in Figure 6, it can be observed that the highest performance is achieved with the hyperspectral-unmixing-based approach, which has an overall performance of 94% sensitivity (95% CI, 0.71–1.00), 85% specificity (95% CI, 0.72–0.93), 87% accuracy (95% CI, 0.77–0.94), 71% MCC, and 92% AUC. The average patch approach has a lower performance with a sensitivity of 88% (95% CI, 0.64–0.99) and specificity of 85% (95% CI, 0.72–0.93). Finally, the center pixel approach has the lowest performance, with a sensitivity of 76% (95% CI, 0.50–0.93) and specificity of 85% (95% CI, 0.72–0.93).

## 4. Discussion

Hyperspectral imaging has the potential to improve breast-conserving surgeries by providing real-time margin assessment, which could enable surgeons to immediately remove additional tissue when necessary to obtain a negative resection margin. As such, supervised classification methods can be employed to develop an algorithm that can determine the presence of tumor tissue within the resection margin. To train supervised classification methods, a dataset with accurate ground-truth labels is required, which is difficult to establish for resection specimens. In this study, we therefore developed a novel approach to assign ground-truth labels to pixels within hyperspectral images based on hyperspectral unmixing. We demonstrated that our novel approach has a higher performance (MCC 0.71) than both the conventional approach to assign the ground-truth label to the center pixel of the inked area (MCC 0.57) or to the average spectrum over all pixels in the inked area (MCC 0.67).

By using this approach, hyperspectral imaging has the potential to outperform other margin assessment techniques that are currently used intraoperatively such as frozen section analysis. Although frozen section analysis has a high diagnostic performance, including a sensitivity of 83% and specificity of 95% [35,36], only a small section of the entire resection surface can be investigated. Furthermore, this technique generally extends the surgery time by 27 min on average [35]. With hyperspectral imaging we are able to image and analyze the entire resection surface of the breast lumpectomy specimen within 10 min (less than 1.5 min per resection side) while the classification performance is comparable to frozen section analysis.

The improved performance of the hyperspectral-unmixing-based approach can be explained using the tumor prediction maps in Figure 4. In the tumor prediction maps of measurements that were assigned a malignant ground-truth label, the location of the highest estimated abundance of malignant tissue is not necessarily in the center of the pixel patch. In practice, it also seems unlikely that histopathologists would be able to slice the tissue with such a high accuracy that the H&E slide would truly correspond to the center of the ink mark. Thus, when ground-truth labels are assigned to the center of the patch, a malignant label might be assigned to a pixel where the spectrum relates to an area that contains almost no tumor tissue. Figure 4 also shows that the tumor hotspot may vary in size. Thus, when ground-truth labels are assigned to the average spectrum of the patch, it might happen that, when the tumor hotspot is relatively small, its corresponding pixels will not contribute accordingly to the total. Consequently, the obtained spectrum predominantly represents healthy tissue, while the ground-truth label from histopathology is malignant. Hence, the mismatch between the labels and measured locations for the center pixel approach as well as the average of the patch approach may have caused the classification model to be trained incorrectly, whereas in the proposed hyperspectral unmixing approach, we selected the pixels that most likely corresponded to the ground-truth labels as input for the training set.

In the current study, we performed hyperspectral unmixing using a linear mixing model. Given the limited number of labeled samples in our dataset, using a linear mixing was more straightforward and advantageous compared to using a nonlinear mixing model. Nevertheless, we acknowledge that a linear mixing model may not be fully adequate to predict the interaction between light and inhomogeneous tissue and thus might result in inaccurate values for the fractional abundances. Even so, the values of the fractional abundances were not the end goal of our approach—they were merely an instrument to select spectra corresponding to ground-truth labels from histopathology. We have shown that this approach outperforms conventional methods to assign ground-truth labels.

The goal of this study was to develop an algorithm to determine the presence of tumor tissue within the resection margin. Thereby, we primarily focused on the discrimination of healthy and malignant tissue without particularly considering the different variants that belong to either of the tissue classes (i.e., healthy; fat and connective tissue; malignant; CIS and IC). As clinical guidelines on re-excisions differ among cancer variants and even subvariants [32,37], the results of the current study might ultimately be insufficient to provide the intraoperative feedback necessary to decide whether or not to perform a re-excision. For example, among CIS, the precursor to invasive carcinoma, a distinction can be made between ductal carcinoma in situ (DCIS) and lobular carcinoma in situ (LCIS). In the USA, a re-excision for DCIS is already indicated when any cells are present within a margin of 2 mm under the resection surface, whereas in the Netherlands this only applies to DCIS cells on the resection surface [32,37]. In addition, the presence of low-grade DCIS may also not indicate a re-excision in all cases. For LCIS, some subvariants are indicative for a re-excision but the classical variant is not. As opposed to CIS, a re-excision for IC is only recommended when the lesion reaches the resection surface and is larger than 4 mm in size [32]. Hence, to provide clinically meaningful feedback to the surgeon, future research should be more focused on the differentiation between the variants of breast cancer. Nevertheless, in uncertain cases the surgeon could still decide to resect some extra tissue during surgery rather than waiting for additional treatment when the resection surface turns out to be tumor-positive. Since the different types of CIS and IC also deviate considerably in terms of cell density [32], it would be worth investigating the influence of their pathological features on the spectral shape.

A common problem in histopathology is the deformation of lumpectomy specimens after surgical removal. When a lumpectomy specimen is placed on a hard surface, flattening of the tissue may occur. Consequently, the specimen might lose almost 50% of its original height [38], which is known as the pancake phenomenon; the up–down margins decrease while the lateral margins increase. As the prior could affect the margin assessment with the risk of inducing false positive results not only during histopathological analysis but also during hyperspectral imaging, the accurate correlation of histology results with the acquired HSI spectra considering the consequences of the pancake effect requires further investigation.

We used ink to mark areas on the surface of the resection specimen that could be identified in the hyperspectral images and could be used to identify a single H&E slide within that area. One downside of the ink marks was that they covered a fairly large area of 5 by 5 mm. Although Kho et al. [9] demonstrated that for the hyperspectral cameras an area larger than 2 by 2 mm should be sufficient to detect tumor tissue in more than 93% of cases, a smaller ink mark would be not practical as it would highly increase the difficulty of tracing back the ink on the corresponding H&E slide. Another downside of the ink marks is that they can disperse between the time when the hyperspectral image is taken and when the H&E slide is made. Although an ink fixator helped to minimize the dispersion of the ink, the marks still slightly blended together in a few cases where the ink marks were placed in close proximity of each other. This increased the difficulty of separating ink marks on the H&E images, adding an uncertainty to the corresponding tissue labels. While this could be prevented by placing the ink marks at a safe distance from each other, placing ink marks close to each other on suspected tumor tissue helped to increase the amount of tumor data in the dataset. Apart from ink, other types of marks could be used to correlate histopathology slides to the hyperspectral image, such as sutures. Regardless, the size of the marker will always be bigger than the thickness of an H&E slide, so our hyperspectral unmixing approach would also be relevant when other marks are used.

Another point to note is that the 0.5 mm resolution (pixel size) of the camera is substantially smaller than the optical diffusion length. The optical diffusion length is a measure of the blurriness of the optical images due to the strong light scattering inside the tissue. This leads to mixing of spectra of adjacent pixels, independent of their ground-truth label. Kho et al. [10] showed that, depending on the wavelength, the information from a diffuse optical spectrum can be smeared out over an area as large as 2 mm. This seems to be in line with our current results, e.g., in the tumor prediction maps shown in Figure 4 we see transitions from 100% healthy to 100% malignant tissue (dark to bright) occurring over roughly 4–5 pixels.

## 5. Conclusions

This is the first study that has used a hyperspectral-unmixing-based approach to determine which pixels should be assigned a ground-truth label from histopathology, and should be used to train a supervised classification algorithm. This approach outperformed conventional approaches to assign ground-truth labels on ex vivo lumpectomy specimens. Since the ultimate goal of our research is to implement hyperspectral imaging into clinical practice, future steps would be to incorporate and classify the entire resection surface of lumpectomy specimens in order to allow real-time feedback during breast-conserving surgery. Furthermore, even though we applied the hyperspectral-unmixing-based approach to breast cancer, it could also be used for other types of cancer, other types of tissue classification, and even outside the medical field to any other field in which hyperspectral imaging is used.

## Figures and Tables

**Table 1:** Patient characteristics.

Characteristic	No. of Patients (%)	Mean ± STD
Age, years		57 ± 11
<50	48 (25)	
50–59	69 (37)	
60–69	43 (23)	
≥70	29 (15)	
Menopausal stage		
Pre	41 (22)	
Peri	16 (8)	
Post	109 (58)	
Unknown	23 (12)	
Breast side		
Left	92 (49)	
Right	97 (51)	
Breast density, ACR ${}^{1}$ score		
1	15 (8)	
2	75 (40)	
3	75 (40)	
4	20 (11)	
Unknown	4 (2)	
Neoadjuvant therapy ${}^{2}$		
Chemotherapy	19 (10)	
Hormone therapy	13 (7)	
Immunotherapy	2 (1)	
None	155 (82)	
Size lumpectomy, cm${}^{3}$		57 ± 56

^1^ American College of Radiology score; 1 = almost entirely fatty. 2 = scattered fibroglandular densities. 3 = heterogeneously dense. 4 = extremely dense. ${}^{2}$ Only patients with either no or partial tumor response to neoadjuvant therapy were included.

**Table 2:** Distribution of tissue types in the lumpectomy dataset.

Tissue Class	Training Set	Test Set
#Patients (#Locations)	Labeled	Labeled
Healthy	129 (221)	35 (53)
Malignant	59 (81)	12 (17)
Total	151 (302)	38 (70)

**Table 3:** Performance metrics of the weighted kNN classifiers for the discrimination of healthy and malignant tissue on the lumpectomy resection surface using different approaches.

	Sensitivity Mean [95% CI]	Specificity Mean [95% CI]	Accuracy Mean [95% CI]	MCC	AUC
Patch center	0.76 [0.50, 0.93]	0.85 [0.72, 0.93]	0.83 [0.72, 0.91]	0.57	0.85
Patch average	0.88 [0.64, 0.99]	0.85 [0.72, 0.93]	0.86 [0.75, 0.93]	0.67	0.93
**Patch unmixing**	**0.94** [0.71, 1.00]	**0.85** [0.72, 0.93]	**0.87** [0.77, 0.94]	**0.71**	**0.92**

## Data Availability

The data presented in this study are available on request from the corresponding author. The data are not publicly available.
